# Could Conservative Iron Chelation Lead to Neuroprotection in Amyotrophic Lateral Sclerosis?

**DOI:** 10.1089/ars.2017.7493

**Published:** 2018-09-10

**Authors:** Caroline Moreau, Véronique Danel, Jean Christophe Devedjian, Guillaume Grolez, Kelly Timmerman, Charlotte Laloux, Maud Petrault, Flore Gouel, Aurélie Jonneaux, Mary Dutheil, Cédrick Lachaud, Renaud Lopes, Grégory Kuchcinski, Florent Auger, Maeva Kyheng, Alain Duhamel, Thierry Pérez, Pierre François Pradat, Hélène Blasco, Charlotte Veyrat-Durebex, Philippe Corcia, Patrick Oeckl, Markus Otto, Luc Dupuis, Guillaume Garçon, Luc Defebvre, Z. Ioav Cabantchik, James Duce, Régis Bordet, David Devos

**Affiliations:** ^1^ Department of Neurology, ALS Center, Lille University, INSERM UMRS_1171, University Hospital Center, LICEND COEN Center, Lille, France.; ^2^ Department of Medical Pharmacology, Lille University, INSERM UMRS_1171, University Hospital Center, LICEND COEN Center, Lille, France.; ^3^ Department of Neuroradiology, Lille University, INSERM UMRS_1171, University Hospital Center, LICEND COEN Center, Lille, France.; ^4^ Department of Preclinical Radiology, Lille University, INSERM UMRS_1171, LICEND COEN Center, Lille, France.; ^5^ Department of Biostatistic, University of Lille, CHU Lille, EA 2694–Santé Publique: épidémiologie et qualité des soins, Lille, France.; ^6^ Department of Pneumology, Lille University, University Hospital Center, Lille, France.; ^7^ Laboratoire d'Imagerie Biomédicale, Sorbonne Universités, UPMC University Paris 06, CNRS, Inserm, Paris, France.; ^8^ Département de Neurologie, AP-HP, Hôpital Pitié-Salpêtrière, Paris, France.; ^9^ Laboratoire de Biochimie, Université François Rabelais, INSERM U930, CHRU, Tours, France.; ^10^ Department of Neurology, Center for Biomedical Research, Ulm University Hospital, Ulm, Germany.; ^11^ INSERM UMR-S1118, Faculté de Médecine de, Strasbourg, France.; ^12^ EA4483 Department of Toxicology, CHU of Lille University, Lille, France.; ^13^ Della Pergola Chair, Alexander Silberman Institute of Life Sciences, Hebrew University, Jerusalem, Israel.; ^14^ Alzheimer's Research UK Cambridge Drug Discovery Institute, University of Cambridge, Cambridge Biomedical Campus, Cambridge, United Kingdom.; ^15^ The Florey Institute of Neuroscience and Mental Health, University of Melbourne, Parkville, Victoria, Australia.

**Keywords:** amyotrophic lateral sclerosis, conservative iron chelator, oxidative stress, neuroprotection, treatment

## Abstract

Iron accumulation has been observed in mouse models and in both sporadic and familial forms of amyotrophic lateral sclerosis (ALS). Iron chelation could reduce iron accumulation and the related excess of oxidative stress in the motor pathways. However, classical iron chelation would induce systemic iron depletion. We assess the safety and efficacy of conservative iron chelation (*i.e.*, chelation with low risk of iron depletion) in a murine preclinical model and pilot clinical trial. In *Sod1*^G86R^ mice, deferiprone increased the mean life span compared with placebo. The safety was good, without anemia after 12 months of deferiprone in the 23 ALS patients enrolled in the clinical trial. The decreases in the ALS Functional Rating Scale and the body mass index were significantly smaller for the first 3 months of deferiprone treatment (30 mg/kg/day) than for the first treatment-free period. Iron levels in the cervical spinal cord, medulla oblongata, and motor cortex (according to magnetic resonance imaging), as well as cerebrospinal fluid levels of oxidative stress and neurofilament light chains were lower after deferiprone treatment. Our observation leads to the hypothesis that moderate iron chelation regimen that avoids changes in systemic iron levels may constitute a novel therapeutic modality of neuroprotection for ALS. *Antioxid. Redox Signal.* 29, 742–748.

## Introduction

Amyotrophic lateral sclerosis (ALS) is characterized by rapid progressive upper and lower motor neuron degeneration, leading to paralysis and death. Iron accumulation has been observed in mouse models and in both sporadic and familial forms of ALS (1, 3–6, 8, 9). Iron accumulation seems to occur at least in microglial cells within motor cortical regions (6) and has been observed in the motor cortex using magnetic resonance imaging (MRI) (1, 6). In patients with sporadic ALS, cerebrospinal fluid (CSF) levels of iron are elevated (4) and elevated serum ferritin levels correlate with shorter survival (9). Importantly, iron chelation has shown therapeutically relevant protective effects in animal models (3, 5). Deferiprone is a unique iron chelator; at low-dose levels, it can cross membranes, decrease regional iron accumulation, redeploy the captured iron to extracellular transferrin, and subsequently distribute iron throughout the body (thus avoiding anemia), defining the “conservative” iron chelation (2).

Innovation The present work provides the first clinical evidence about the neuroprotective potential of a therapeutically safe chelation treatment on early-stage amyotrophic lateral sclerosis (ALS) patients, which responded significantly to treatment in both brain iron deposits and indicators of disease progression. The novel treatment relied on oral administration of deferiprone that by chelation of labile iron it conferred on oxidation-stressed animals, improved motor functions, while essentially sparing systemic iron. The paradigmatic modality of chelation with deferiprone in ALS has prompted a multicenter study.

## Dose and Sex Effect on Neuroprotection with Deferiprone in the Murine Model of ALS

**FIG. 1.**
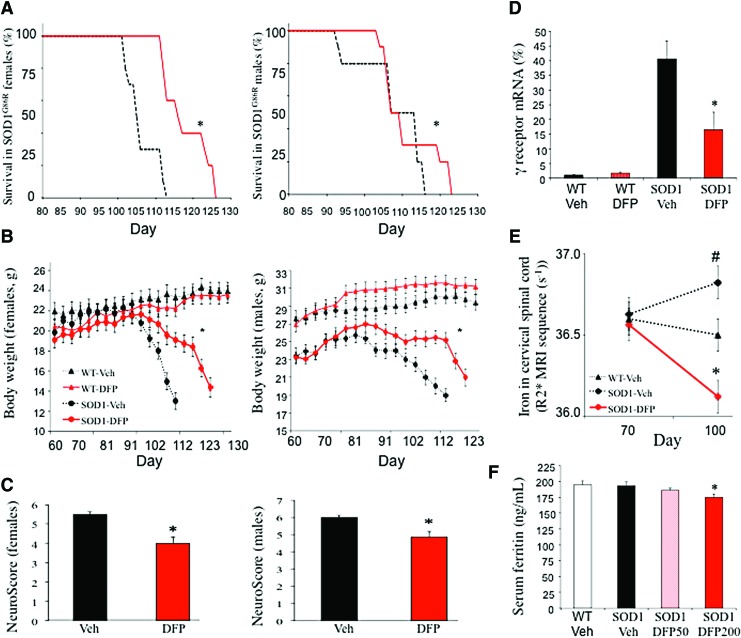
**Pharmacological effects of DFP in a murine model of ALS**. Data are mean ± SEM in all experiments and doses, *n* = 10 per group for each sex and each dose. For the females, the best dose was 50 mg/kg/day, the dose of 100 mg/kg/day was less effective, and the dose of 200 mg/kg/day was not inefficient. For the males, the only efficient dose was 200 mg/kg/day. All the figures are presented with the dose of 50 mg/kg/day for the females and 200 mg/kg/day for the males. **(A)** The survival rates were significantly improved for female (in *red*) and male (in *black*) SOD1^G86R^ mice (from disease and treatment onset onward) in the DFP group (*solid line*) and the vehicle group (Veh, *dotted line*) (log-rank test; females: *p* = 0.011; males: *p* = 0.03). **(B)** Change in body weight in female and male SOD1^G86R^ mice from disease and treatment onset until death, in four groups: WT-Veh (*black triangles* with a *dotted line*), WT-DFP (*red triangles* with a *solid line*), SOD1^G86R^-Veh (*black circles* with a *dotted line*), and SOD1^G86R^-DFP (*red circles* with a *solid line*) (ANCOVA adjusted on baseline, *p < 0.05 *vs.* untreated SOD1^G86R^ mice). **(C)** The peak NeuroScore (a quick phenotypic neurological scoring system for evaluating disease progression in the mouse model of ALS: 0: no impairment; 6: greatest possible impairment) in the SOD1^G86R^-Veh group (*black bar*) and the SOD1^G86R^-DFP group (*red bar*) (Mann–Whitney test, *p < 0.05 *vs.* untreated SOD1^G86R^ mice). **(D)** As measured by qRT-PCR, acetylcholine receptor subunit γ receptor RNA expression as a percentage (%) of the control value in the *left* gastrocnemius muscle in SOD1^G86R^ female mice treated with 50 mg/kg/day DFP (Mann–Whitney test, *p < 0.05 *vs*. untreated SOD1^G86R^ mice). **(E)** Magnetic resonance imaging of the cervical spinal cord at day 70 (*i.e.*, just before the appearance of symptoms and treatment onset) and at day 100 (*i.e.*, with marked motor impairments before death) in untreated female SOD1^G86R^ mice (*triangles* with a *black solid line*), WT female mice (*squares* with a *dotted line*), and SOD1^G86R^ female mice treated with 50 mg/kg/day DFP (*red line*). R2* ( = 1/T2*) was obtained in a manually drawn region of interest using a monoexponential fitting of the signal decay with the echo time (Kruskal–Wallis test *p < 0.05 *vs*. untreated SOD1^G86R^ mice, ^#^*p* < 0.05 *vs*. WT untreated mice). **(F)** Serum ferritin levels (ng/mL) from serum obtained at end of treatment: not significantly reduced after 50 mg/kg/day and 100 mg/kg/day (data not shown), but significantly reduced after 200 mg/kg/day (Kruskal–Wallis test *p < 0.05 *vs*. untreated SOD1^G86R^ mice). ALS, amyotrophic lateral sclerosis; DFP, deferiprone; SOD, superoxide dismutase; WT, wild type. To see this illustration in color, the reader is referred to the web version of this article at www.liebertpub.com/ars

A dose- and sex-dependent effect of deferiprone on survival was observed in the SOD, superoxide dismutase (SOD)1^G86R^ mouse model (Fig. 1). The female mice in the 50 mg/kg/day deferiprone groups survived for 13 days longer than those in the vehicle group (Fig. 1A). This corresponded to a 56% extension in survival from disease onset (defined as the peak in body weight) (Fig. 1B). The dose of 100 mg/kg/day was less effective and the dose of 200 mg/kg/day was not effective (not shown). A significant effect was observed in male mice only with the highest dose (200 mg/kg/day). Deferiprone improved the animals' physical examination, as shown by greater body weight (Fig. 1B), a lower peak in neurological impairment (*i.e.*, a lower Neuroscore, a quick phenotypic neurological scoring system) (Fig. 1C), and return of gene expression of *acetylcholine receptor subunit γ (Chrng)* to wild-type levels (marker of denervation of muscles) (Fig. 1D). Importantly, treated mice had less iron accumulation in the spinal cord (shown by MRI T2* sequence) compared with vehicle control mice (Fig. 1E), demonstrating an ability of deferiprone to hit the biological target. As with treatment in other neurological models (2), deferiprone did not induce anemia, and serum ferritin was only marginally below normal levels at the highest dose (Fig. 1F).

## Safety Profile of Deferiprone in Early ALS Patients

Twenty-three consecutive sporadic patients were enrolled (22 limb onset and 1 bulbar onset) (Table 1). Four patients dropped out: one patient died after a fall, and 3 withdrew their consent. All were compliant to medication. The non-neurological physical examination remained unchanged. All patients displaying normal hematologic profiles, a slight elevation in urine iron levels, and a transient decrease in serum ferritin (in the normal ranges) was observed in the first 3 months of treatment.

**Table 1.** Neurological and Systemic Parameters in Amyotrophic Lateral Sclerosis Patients at Inclusion and with up to 12 Months of Deferiprone Treatment

	*← The SAFE-FAIR-ALS trial (months) →*						
	*Baseline*	*V3 3 months of no treatment*	*V6 3 months of DFP*	p*-value for V3* vs*. V6*	*V9 6 months of DFP*	*V12 9 months of DFP*	*V15 12 months of DFPFP*
Population (*n* = 23)	Mean ± SD age: 56.2 ± 9.8 years; 4 female/13 male; mean ± SD disease duration (since first signs): 11.7 ± 5.7 months						
Drop out: 4 patients: 1 death related to brain hemorrhage after a fall and 3 withdrew their consent							
Adverse events during V3–V18 (*n* = 23)							
Falls with or without trauma (*n* = 9); respiratory failure with or without bronchitis (*n* = 4); arterial hypertension (*n* = 2); transient chest pain (*n* = 2); transient abdominal pain (*n* = 1); pulmonary embolism (*n* = 1); atrial fibrillation (*n* = 1); arterial thrombosis (*n* = 1); transient nausea (*n* = 1); transient postural tremor (*n* = 1); pruritus (*n* = 1); gastritis (*n* = 1)							
Handicap test (*n* = 19)							
ALSFRS-R	**40.5 ± 4** **41 [36–45]**	**35.6 ± 7** **36 [31–42]**	**33.1 ± 8** **33 [26–41]**	0.013^*^	31.3 ± 9 31 [26–41]	29.6 ± 11 31 [20–40]	29 ± 11 35 [20–39]
BMI	**26.3 ± 4** **25 [24–28]**	**25.9 ± 4** **26 [23–28]**	**26.0 ± 4** **25 [24–28]**	0.047^**^	26.4 ± 4 26 [24–28]	26.9 ± 3 27 [24–28]	25 ± 3 24 [24–27]
MMT	93.9 ± 7 92 [89–99]	88.0 ± 11 90 [81–95]	79.4 ± 13 78 [73–90]	0.33^**^	77.0 ± 16 75 [68–88]	75.2 ± 17 77 [66–84]	72 ± 14 72 [66–79]
SVC	102.6 ± 18 102 [89–112]	95.3 ± 20 90 [79–110]	87.3 ± 25 90 [60–96]	0.1^**^	79.8 ± 23 83 [67–96]	78.8 ± 31 74 [57–96]	75 ± 35 76 [58–92]
Iron accumulation on MRI: R2^*^ (s^−1^) (*n* = 19)							
Precentral-central cortex		14.9 ± 1.3	**15.6 ± 1.4**	**14.3 ± 1.6**	0.038^**^ (V3–V6)	13.3 ± 2	
Medulla oblongata		16.2 ± 3	**17.4 ± 2**	**15.5 ± 1**	0.047^**^ (V3–V6)	15.7 ± 2	
Cerebellum		16.9 ± 1	17.1 ± 1	16.9 ± 1	0.3^**^ (V3–V6)	17.1 ± 1	
Cervical spinal cord		**40.2 ± 5.6**	**42.2 ± 5.6**	**39.2 ± 4.8**	0.002^**^(V3–V6)	39 ± 4.9	
Ferritin and iron in body fluids (*n* = 19)							
Ferritin		134 [100–175]	**143 [89–193]**	**134 [72–170]**	**0.002 (V3–V6)**	150 [99–206]	160 [101–220]
Ferritin (CSF)		—	**12.3 [10–16]**	—	**0.027 (V3–V9)**	**9.7 [9–14]**	
Iron (urine)		11.4 [4–19]	**12.4 [8–12]**	**98.6 [32–99]**	**0.001 (V3–V6)**	85 [42–122]	72 [21–99]
Oxidative stress marker in CSF (*n* = 19)							
8-OHdG		—	**6 [5.4–6.7]**	—	**0.029 (V3–V9)**	**5.2 [5–6.5]**	—
SOD-1 in CSF (*n* = 19)							
SOD		—	0.6 [0.5–0.7]	—	0.3 (V3–V9)	0.6 [0.5–0.7]	—
Neurofilaments in CSF (*n* = 19)							
NfL		—	5698 [3332–9599]	—	**0.08 (V3–V9)**	3492 [2807–6288]	
pNfH		—	2578 [1338–3245]	—	0.3 (V3–V9)	2381 [1361–3255]	—

Data are mean ± standard deviation (first line) and median value [Quartile 1-Quartile 3] (second line). A revised ALSFRS-R score was measured as the primary outcome in the patient cohorts, whereas BMI, muscle strength (measured by MMT), and SVC were also evaluated as secondary outcomes (Student's test (^*^) or Wilcoxon test (^**^), depending on the distribution assessed using histograms and the Shapiro–Wilk test).

Iron content was quantified by R2^*^ transverse relaxation rates ( = 1/T2^*^) measured using a 3-Tesla MRI system (analysis of variance for repeat measures; the statistically significant contrast analyses for V0 *vs*. V3, V3 *vs*. V6, and V6 *vs*. V9 are indicated).

Additional secondary biological outcomes were evaluated using previously characterized assays. Units observed in control patients were within the established ranges previously reported. Ferritin in CSF and serum in ng/mL. Iron in urines in μg/24 h. 8-oxdG: DNA adduct of 8-oxo-7,8-dihydro-2′-deoxyguanosine in ng/mL (Highly Sensitive 8-OHdG Check, Gentaur France SARL, Paris, France), SOD in U/mL (whole blood, as published elsewhere^8^); NfL: neurofilament light chain in pg/mL (Simoa/Digital ELISA platform) and pNfH: phosphorylated neurofilament heavy chain in pg/mL (BioVendor kit) (Student's *t*-test (^*^) or Wilcoxon test (^**^), depending on the distribution assessed using histograms and the Shapiro–Wilk test).

The bold values are statistically significant.

ALS, amyotrophic lateral sclerosis; ALSFRS-R, ALS Functional Rating Scale; BMI, body mass index; CSF, cerebrospinal fluid; DFP, deferiprone; MMT, manual muscle testing; MRI, magnetic resonance imaging; SVC, slow vital capacity; SOD, superoxide dismutase.

## Is There a Disease Modifying Effect of Deferiprone in Early ALS Patients?

The decrease in the ALS Functional Rating Scale (ALSFRS-R) score was significantly smaller for the first 3 months of deferiprone treatment than for the 3-month treatment-free period (*p* = 0.013) (Table 1). Likewise, the decrease in the body mass index (BMI) was significantly different, with a decrease during the first 3 months and a small increase during the treatment period (no percutaneous endoscopic gastrostomy feeding) (*p* = 0.047). Then, the BMI remained unchanged for 9 months. The reduction in manual muscle testing (MMT) scores was lower in patients on deferiprone than in matched patients from the Mitotarget study, although this difference did not reach statistical significance (*p* = 0.09) (Table 2).

**Table 2.** Comparisons of 9 Months of Disease Progression in Patients on Deferiprone *Versus* a Matched Population from the Mitotarget Trial

*Parameters and populations*	*Deferiprone treatment*	*Three months of DFP treatment*	*Nine months of DFP treatment*	p^a^
ALSFRS				
SAFE-FAIR, mean (SD)	35.6 (6.7)	33.1 (8.2)	30.6 (11.2)	0.50
Median (IQR)	34.0 (30.0–43.0)	33.0 (26.0–41.0)	(35.0) (20.0–41.0)	
Mitotarget, mean (SD)	35.4 (8.4)	32.8 (9.9)	30.6 (10.5)	
Median (IQR)	38.0 (30.0–42.0)	35.5 (26.5–41.0)	35.5 (23.0–38.0)	
BMI				
SAFE-FAIR, mean (SD)	26.0 (4.5)	26.1 (4.9)	27.4 (2.9)	0.32
Median (IQR)	25.7 (23.6–28.4)	25.5 (24.0–29.0)	27.4 (25.2–28.9)	
Mitotarget, mean (SD)	23.8 (3.2)	23.2 (3.5)	23.0 (3.6)	
Median (IQR)	23.2 (21.4–25.7)	21.9 (20.7–25.3)	22.5 (20.5–25.5)	
MMT				
SAFE-FAIR, mean (SD)	88.1 (11.8)	79.4 (13.3)	76.0 (17.9)	**0.090**
Median (IQR)	90.5 (80.0–96.0)	78.5 (73.0–90.0)	78.5 (68.5–85.5)	
Mitotarget, mean (SD)	118.8 (26.4)	112.8 (29.7)	97.5 (39.0)	
Median (IQR)	127.0 (110.0–139.0)	121.5 (86.5–135.0)	111.0 (56.0–122.0)	
SVC				
SAFE-FAIR, mean (SD)	96.3 (20.2)	89.4 (24.1)	82.9 (28.3)	0.97
Median (IQR)	91.6 (80.4–113.0)	90.4 (66.7–102.8)	80.7 (61.8–105.4)	
Mitotarget, mean (SD)	92.1 (20.2)	84.7 (23.7)	76.4 (36.4)	
Median (IQR)	92.5 (78.0–106.0)	88.0 (75.0–98.0)	75.4 (49.5–107.5)	

The revised ALSFRS-R, BMI, muscle strength (measured by MMT), and SVC were evaluated.

Model for ALSFRS: ${ \rm{ALSFR}}{{ \rm{S}}_{{ \rm{ik}}}} = {{ \rm{ \beta }}_0} + {{ \rm{ \beta }}_1}{{ \rm{t}}_{6{ \rm{ik}}}} + {{ \rm{ \beta }}_2}{{ \rm{t}}_{12{ \rm{ik}}}} + {{ \rm{ \beta }}_3}{ \rm{Grou}}{{ \rm{p}}_{{ \rm{ik}}}} + {{ \rm{ \beta }}_4}{ \rm{Grou}}{{ \rm{p}}_{{ \rm{ik}}}}{ \rm{*}}{{ \rm{t}}_{6{ \rm{ik}}}} + {{ \rm{ \beta }}_5}{ \rm{Grou}}{{ \rm{p}}_{{ \rm{ik}}}}{ \rm{*}}{{ \rm{t}}_{12{ \rm{ik}}}} + {{ \rm{ \beta }}_6}{ \rm{ALSFR}}{{ \rm{S}}_{{ \rm{baselin}}{{ \rm{e}}_{ \rm{i}}}}} + {{ \rm{ \gamma }}_{0{ \rm{i}}}} + {{ \rm{ \gamma }}_{1{ \rm{k}}}} + {{ \rm{ \varepsilon }}_{{ \rm{ik}}}}$. ${ \rm{ik}}$ = for any subject i, for any bloc k, ${{ \rm{ \gamma }}_{0{ \rm{i}}}} + {{ \rm{ \gamma }}_{1{ \rm{k}}}}$ are random effects.

The bold value is statistically significant.

^a^ *p*-Value for the overall interaction between *group* and *time*, adjusted for the outcome level before DFP treatment (baseline).

## Iron Accumulation and Oxidative Stress Reduction Under Deferiprone in Early ALS Patients

A significant decrease in iron concentration (shown by a decrease in R2*) was observed in the cervical spinal cord, the medulla oblongata, and the motor cortex but not in areas outside the motor system (*i.e.*, the cerebellum and the occipital cortex) following treatment with deferiprone. Iron levels, oxidative stress markers, and neurofilament light chains were lower after deferiprone treatment in the CSF (Table 1).

## Conservative Iron Chelation

The present study is a first to demonstrate the safety of conservative iron chelation in ALS. In both a murine model of familial ALS and sporadic ALS patients, low-dose deferiprone was associated with a decrease in pathologic iron accumulation in the central motor pathways but did not alter iron metabolism in other regions of the brain or in the periphery. This new therapeutic strategy appears to maintain the patient's overall aerobic metabolism and limit excess oxidative stress—as has been observed in Parkinson's disease (2).

Deferiprone significantly increased survival, as previously reported with other iron chelators (3, 5). This was observed despite treatment initiation at the symptomatic stage in the phenotypically aggressive SOD1^G86R^ model. An interaction between dose and sex was observed; disease severity, iron accumulation, and the required dose were higher in males than in females.

Deferiprone had a good safety profile in patients, with adverse events mostly relating to persistent ALS symptoms. We did not detect any of the adverse events occasionally observed in patients, with systemic iron overload, treated with 100 mg/kg/day deferiprone.

Encouragingly, for a safety trial with a small number of patients, deferiprone treatment was associated with slower disability progression and weight loss. However, the occurrence of a nocebo effect and then a placebo effect cannot be ruled out, and so, a large, multicenter, double-blind, placebo-controlled, randomized clinical trial is underway.

## Materials and Methods

### SOD1^86R^ transgenic mice

All animal experiments were carried out in accordance with the “Principles of Laboratory Animal Care,” the current French and European Union legislative and regulatory framework (APAFIS#4269-2015112317225759), and the European ALS group's preclinical trial guidelines of 2010. A dose/response study was performed in FVB-Tg(Sod1*G86R)M1Jwg/J mice (JAX Laboratories) with 50, 100, or 200 mg/kg p.o. deferiprone or vehicle twice a day (10 in each group). Study treatment was initiated at 75 days, that is, an age at which these mice are devoid of motor symptoms but already present with weight loss. The investigators were blinded to the study treatment. MRI of the cervical spinal cord was done using a 7-T MR system (BioSpec Bruker, Ettlingen, Germany), using a multiecho T2*-weighted sequence (number of echoes: 12; first echo time: 4 ms; echo spacing: 7 ms; repetition time: 1500 ms; slice thickness: 1 mm; field of view: 200 × 250 mm; matrix: 256 × 256; number of signal averages: 2).

### Clinical trial

A single-center, single-arm, 12-month pilot clinical trial was performed to evaluate the effect of deferiprone on patients with ALS. The patients were followed for a 3-month treatment-free period and then treated for 12 months with the liquid formulation of deferiprone at a dose level of 30 mg/kg/day (morning and evening dose). The patients were recruited between December 2013 and January 2015, and all provided written informed consent. All patients had been taking riluzole. Treatment compliance (>80%) was assessed by questioning the participants and inspection of the dispensed packs of medication. The primary outcome criterion was disease progression, measured using the revised ALSFRS-R. The 3-month treatment-free period was compared with the first 3 months of treatment. The secondary outcomes included MMT, BMI, slow vital capacity, and CSF levels of markers for oxidative stress and neurofilaments. Physical examination was assessed every 3 months together with adverse event reports and reviewed anonymously by an independent safety monitoring board. Weekly blood counts were used to monitor the risk of neutropenia. For exploratory purposes, 19 patients treated with deferiprone for 9 months were compared with 19 matched individuals from among the 512 patients in the Mitotarget trial (negative results with olesoxime; NCT:00868166) (7).

Iron content was quantified by R2* transverse relaxation rates ( = 1/T2*) measured using a 3-Tesla MRI system (Achieva, Philips Medical Systems, Best, The Netherlands), using a 2D fast-field echo multiecho sequence (number of echoes: 15; first echo time: 3.6 ms; echo spacing: 3.3 ms; repetition time: 1803 ms). Two stacks were subsequently acquired in the axial plane; 17 slices for each (slice thickness: 2 mm; isotropic, no gap, field of view: 230 × 190 mm; matrix: 116 × 95; number of signal averages: 2) to cover a volume between the floor of the fourth ventricle and the corpus callosum convexity. Images were processed using a T2* tool on an IDL virtual machine (V2, www.rsinc.com/IDL). A monoexponential signal decay with the echo time was obtained by voxel-by-voxel nonlinear least-squares fitting of the multiecho data [S(t) = S_0_.e^−t.R2*^; where t = echo time, S = measured signal, R2* = transverse relaxation rate]. Regions of interest were manually drawn on R2* maps by the same operator, who was blinded to the clinical data.

### Statistical analysis

Differences in main outcomes between the treatment-free period (months 0–3) and the first three months of deferiprone treatment (months 3–6) were assessed with a paired T test (for normally distributed variables) or Wilcoxon signed-rank test.

Taking into account the differences in baseline characteristics between the patients on deferiprone and the Mitotarget population, we performed 1:1 matching on three prespecified factors: age (±5 years), disease duration (±2 months), and sex. Changes in the main outcomes between month 3 and 15 (9 months of treatment) in the paired groups were compared using linear mixed models with random coefficients. *Group*, *time*, the *group* × *time* interaction, and the baseline value were considered as fixed effects, with the participant and block matching considered as random effects. All statistical tests were two sided (*p* < 0.05), and all data were analyzed using SAS software (version 9.4, SAS Institute, Inc., Cary, NC).

### Study approval

All clinical investigations were performed in accordance with the tenets of the Declaration of Helsinki. All patients provided written informed consent to participation. A local institutional review board approved the aims and procedures of the main study (national reference number: 2013-001228-21; ClinicalTrials.gov reference: NCT02164253) and a compassionate 12-month extension. The study and the article followed the consort statement.

## Abbreviations Used

ALS: amyotrophic lateral sclerosis
ALSFRS-R: ALS Functional Rating Scale
BMI: body mass index
CSF: cerebrospinal fluid
DFP: deferiprone
MMT: manual muscle testing
MRI: magnetic resonance imaging
SCV: slow vital capacity
SOD: superoxide dismutase

## References

[1] Adachi Y 1, Sato N, Saito Y, Kimura Y, Nakata Y, Ito K, Kamiya K, Matsuda H, Tsukamoto T, and Ogawa M Usefulness of SWI for the detection of iron in the motor cortex in amyotrophic lateral sclerosis. J Neuroimaging 25: 443–451, 2015 24888543 10.1111/jon.12127

[2] Devos D, Moreau C, Devedjian JC, Kluza J, Petrault M, Laloux C, Jonneau X, Ryckewaert G, Garçon G, Rouaix N, Duhamel A, Jissendi P, Dujardin K, Auger F, Ravasi L, Hopes L, Grolez G, Firdaus W, Sablonnière B, Strubi-Vuillaume I, Zahr N, Destée A, Corvol JC, Pöltl D, Leist M, Rose C, Defebvre L, Marchetti P, Cabantchik ZI, and Bordet R Targeting chelatable iron as a therapeutic modality in Parkinson's disease. Antioxid Redox Signal 21: 195–210, 2014 24251381 10.1089/ars.2013.5593 PMC4060813

[3] Golko-Perez S, Amit T, Bar-Am O, Youdim MB, and Weinreb O A novel iron chelator-radical scavenger ameliorates motor dysfunction and improves life span and mitochondrial biogenesis in SOD1^G93A^ ALS Mice. Neurotox Res 31: 230–244, 2017 27826939 10.1007/s12640-016-9677-6

[4] Ignjatović A 1, Stević Z, Lavrnić D, Nikolić-Kokić A, Blagojević D, Spasić M, and Spasojević I Inappropriately chelated iron in the cerebrospinal fluid of amyotrophic lateral sclerosis patients. Amyotroph Lateral Scler 13: 357–362, 2012 22424123 10.3109/17482968.2012.665929

[5] Jeong SY, Rathore KI, Schulz K, Ponka P, Arosio P, and David S Dysregulation of iron homeostasis in the CNS contributes to disease progression in a mouse model of amyotrophic lateral sclerosis. J Neurosci 29: 610–619, 2009 19158288 10.1523/JNEUROSCI.5443-08.2009 PMC6665152

[6] Kwan JY, Jeong SY, Van Gelderen P, Deng HX, Quezado MM, Danielian LE, Butman JA, Chen L, Bayat E, Russell J, Siddique T, Duyn JH, Rouault TA, and Floeter MK Iron accumulation in deep cortical layers accounts for MRI signal abnormalities in ALS: correlating 7 tesla MRI and pathology. PLoS One 7: e35241, 2012 22529995 10.1371/journal.pone.0035241 PMC3328441

[7] Lenglet T, Lacomblez L, Abitbol JL, Ludolph A, Mora JS, Robberecht W, Shaw PJ, Pruss RM, Cuvier V, and Meininger V; Mitotarget Study Group. A phase II-III trial of olesoxime in subjects with amyotrophic lateral sclerosis. Eur J Neurol 21: 529–536, 2014 24447620 10.1111/ene.12344

[8] Lu A, Rajanala M, Mikkilineni S, Cahill S, Brown C, Berry RD, and Rogers J The 5’-untranslated region of the C9orf72 mRNA exhibits a phylogenetic alignment to the cis-aconitase iron-responsive element; novel therapies for amytrophic lateral sclerosis. Neurosci Med 7: 15–26, 2016

[9] Veyrat-Durebex C, Corcia P, Mucha A, Benzimra S, Mallet C, Gendrot C, Moreau C, Devos D, Piver E, Pagès JC, Maillot F, Andres CR, Vourc'h P, and Blasco H Iron metabolism disturbance in a French cohort of ALS patients. Biomed Res Int 485723, 2014 25101285 10.1155/2014/485723 PMC4101961

